# Evaluation of Cardiac Involvement in Children with Dengue by Serial Echocardiographic Studies

**DOI:** 10.1371/journal.pntd.0003943

**Published:** 2015-07-30

**Authors:** Tawatchai Kirawittaya, In-Kyu Yoon, Sineewanlaya Wichit, Sharone Green, Francis A. Ennis, Robert V. Gibbons, Stephen J. Thomas, Alan L. Rothman, Siripen Kalayanarooj, Anon Srikiatkhachorn

**Affiliations:** 1 Queen Sirikit National Institute of Child Health, Bangkok, Thailand; 2 Department of Virology, Armed Forces Research Institute of Medical Sciences, Bangkok, Thailand; 3 Division of Infectious Diseases and Immunology, Department of Medicine, University of Massachusetts Medical School, Worcester, Massachusetts, United States of America; 4 United States Army Institute of Surgical Research, Institute of Surgical Research, Houston, Texas, United States of America; 5 Viral Diseases Branch, Walter Reed Army Institute of Research, Silver Spring, Maryland, United States of America; 6 Institute for Immunology and Informatics, University of Rhode Island, Providence, Rhode Island, United States of America; Oregon Health and Science University, UNITED STATES

## Abstract

**Background:**

Infection with dengue virus results in a wide range of clinical manifestations from dengue fever (DF), a self-limited febrile illness, to dengue hemorrhagic fever (DHF) which is characterized by plasma leakage and bleeding tendency. Although cardiac involvement has been reported in dengue, the incidence and the extent of cardiac involvement are not well defined.

**Methods and Principal findings:**

We characterized the incidence and changes in cardiac function in a prospective in-patient cohort of suspected dengue cases by serial echocardiography. Plasma leakage was detected by serial chest and abdominal ultrasonography. Daily cardiac troponin-T levels were measured. One hundred and eighty one dengue cases were enrolled. On the day of enrollment, dengue cases that already developed plasma leakage had lower cardiac index (2695 (127) vs 3188 (75) (L/min/m^2^), p = .003) and higher left ventricular myocardial performance index (.413 (.021) vs .328 (.026), p = .021) and systemic vascular resistance (2478 (184) vs 1820 (133) (dynes·s/cm^5^), p = .005) compared to those without plasma leakage. Early diastolic wall motion of the left ventricle was decreased in dengue cases with plasma leakage compared to those without. Decreased left ventricular wall motility was more common in dengue patients compared to non-dengue cases particularly in cases with plasma leakage. Differences in cardiac function between DF and DHF were most pronounced around the time of plasma leakage. Cardiac dysfunction was transient and did not require treatment. Transient elevated troponin-T levels were more common in DHF cases compared to DF (14.5% vs 5%, p = 0.028).

**Conclusions:**

Transient left ventricular systolic and diastolic dysfunction was common in children hospitalized with dengue and related to severity of plasma leakage. The functional abnormality spontaneously resolved without specific treatment. Cardiac structural changes including myocarditis were uncommon.

## Author Summary

Dengue is a viral infection with a wide range of symptoms from a self-limiting fever called dengue fever (DF) to dengue hemorrhagic fever (DHF) which is characterized by leaky blood vessels and bleeding that can lead to shock in severe cases. Abnormal heart function has been reported but the frequencies and the progression of heart involvement are not well defined. In this study children with dengue had serial evaluation of their heart function during the course of the illness. Patients with DHF had comparatively low blood volume at the time of fever resolution and had decreased blood flow into the left lower heart chamber compared to DF cases. Relaxation and contraction of the left side of the heart were also relatively decreased in DHF. These abnormalities may contribute to the clinical response and complications of fluid replacement in dengue.

## Introduction

Infection by dengue viruses (DENV) results in clinical presentation ranging from asymptomatic infection to a fatal viral hemorrhagic fever characterized by plasma leakage and bleeding [1–3]. Plasma leakage in dengue occurs around the time of defervescence, is localized primarily in the serosal cavities, and usually lasts approximately 48–72 hours. Severe plasma leakage can lead to shock (dengue shock syndrome). Although the principal mechanism of shock is due to decreased intravascular volume, abnormal cardiac function may contribute to cardiovascular compromise. Several studies have reported cardiac involvement in dengue including myocarditis and heart failure [4–10]. Functional impairment and electrocardiographic abnormalities have also been described [11–14]. The incidence and severity of cardiac involvement in dengue has varied between studies, likely as a result of differences in study design, methodology, and populations.

Early detection of plasma leakage and fluid replacement is critical in the treatment of dengue. It has been observed that excessive fluid treatment can lead to pulmonary edema in some cases [15–17]. Increased intravascular volume due to fluid intake and reabsorption of fluid from serosal cavities has been thought to be the underlying mechanism. However, it remains possible that abnormalities in cardiac function may also contribute to pulmonary edema.

To further our understanding of cardiac involvement in dengue, we undertook serial echocardiographic studies of cardiac function in dengue cases. We characterized the dynamics of cardiac functional indices and analyzed them in the context of the clinical course and the extent of plasma leakage. Our findings indicate that myocarditis is uncommon, but transient functional changes are common and correlate with the extent of plasma leakage. Diastolic dysfunction characterized by impaired left ventricle (LV) relaxation was the most prominent abnormality. These findings have practical implications for fluid management in dengue.

## Methods

### Ethics statement

The study was approved by the Institutional Review Boards of the Thai Ministry of Public Health, the Walter Reed Army Institute of Research Institutional Review Board. Written informed consent was obtained from the parent or the legal guardian of each study subject.

### Patients

Children less than 15 years of age who were hospitalized for suspected dengue at Queen Sirikit National Institute of Child Health (QSNICH) in 2010 to 2012 were enrolled. Criteria for suspected dengue included cases with febrile illness without an obvious focal source of infection and with compatible laboratory findings including leucopenia or thrombocytopenia. Patients with chronic hematologic or immunologic conditions were excluded. Patients were treated according to World Health Organization (WHO) guidelines [2]. The patients were encouraged to drink. Intravenous fluid was administered in cases with dehydration and inadequate oral fluid intake. The rate and amount of fluid were adjusted according to the clinical status and the severity of dehydration. In shock cases, 10 ml/kg fluid was given as bolus or within one hour for resuscitation and the rate of fluid was adjusted subsequently according to clinical status following an established treatment guideline at QSNICH. DENV infections were confirmed by RT-PCR to detect viral RNA in plasma obtained on the day of study enrollment and by serology of paired acute and convalescent plasma [18, 19] Daily complete blood count, plasma albumin and serum aspartate and alanine aminotransferase (AST, ALT) levels were obtained. Echocardiographic and chest and abdominal ultrasonographic studies were performed daily.

Cases with negative dengue serology were classified as non-dengue febrile illness. Confirmed dengue cases were assigned as dengue fever (DF) or dengue hemorrhagic fever (DHF) according to the 1997 WHO case definitions [2]. DHF cases were graded according to severity as grades I-IV. Cases were also classified as dengue and severe dengue according to the 2009 WHO guidelines[3]. Severe dengue was defined as cases with 1) plasma leakage requiring fluid resuscitation from shock, 2) significant bleeding (defined as cases that required blood transfusion in this study), and 3) evidence of organ failure including AST or ALT >1000 IU/ml. Case classification was performed after the completion of the study by investigators not involved in patient care.

The day of defervescence (temperature <38°C) was defined as fever day 0. Days before and after defervescence were defined as fever day –1, -2, and +1, +2, etc. The study was approved by the Institutional Review Boards of the QSNICH, Thai Ministry of Public Health, and the Walter Reed Army Institute of Research. Written informed consent was obtained from the legal guardian of each participant. Assent was obtained from children who were at least 7 years old.

### Cardiac enzymes

Daily plasma samples obtained during hospitalization and at the early convalescence follow up visit (approximately 5 days after hospital discharge) were assayed for troponin-T levels using the Elecsys Troponin T hs assay (Roche Diagnostics, Elcsys, Germany). Levels >30 pg/ml were considered elevated. Plasma samples collected on the first day of hospitalization and at the early convalescence visit were also analyzed for creatine kinase MB (CPK-MB) isoenzyme levels by ELISA (MyBioSource, U.S.A.). The assay range was 0.2–60 ng/ml. All tests were performed in batch after study completion and without the knowledge of the clinical diagnosis.

### Ultrasonography

Daily ultrasonography was performed as previously described [20]. The vertical dimensions of pleural effusions were measured as the distance between the top of the dome of the diaphragm and the base of the lung visualized by upright midaxillary longitudinal scans of the right hemithorax. The dimensions of ascitic fluids in the perivesicular area were measured in the transverse scan of the lower abdomen.

### Echocardiography

Daily echocardiography was performed by a single cardiologist (TK) using a CX50 CompactXtreme ultrasound system (Philips Healthcare). All measurements were obtained on a daily basis without knowledge of the diagnostic laboratory results. Systolic and diastolic blood pressures and electrocardiograms were recorded during the examinations. Routine 2-D echocardiogram and color flow Doppler were obtained in subcostal and apical 4-chamber views. An M-mode scan of the LV obtained from a standard parasternal long-axis view, at the level of the mitral valve (MV) tip, was recorded simultaneously with the electrocardiogram. Measurements of LV walls and dimensions were performed in accordance with published guidelines [21, 22]. Transmitral pulsed-wave Doppler velocities (peak E- and A-wave velocities) were measured in the apical four chamber view with the sample volume positioned at the MV. Tissue Doppler Imaging (TDI) of the LV was performed using pulsed wave Doppler assessment of the medial and lateral MV annulus. Peak tissue medial and lateral S-wave (S), E-wave (Ea) and A-wave (Aa) velocities were measured. Myocardial performance index (MPI) was calculated from TDI of the LV using the following formula: (isovolumic contraction time + isovolumic relaxation time)/ejection time). Blood pressure was measured by oscillometric method at the time of echocardiography. An average value from at least three consecutive measurements was calculated. TDI parameters were assessed using published age-specific normal values [23]. Values below the 5^th^ or above the 95^th^ percentile of normal values were deemed abnormal.

The inferior vena cava (IVC) diameter was measured using the subcostal view, below the level of the hepatic veins. Pericardial effusions, abnormal electrocardiograms, and other anatomical and functional findings were recorded when present.

### Statistical methods

Variables are reported as mean (SE) or number (%) as appropriate. Analysis of categorical variables was performed using Chi square. Continuous variables were tested with Shapiro-Wilk’s tests for normality of distribution. Normally distributed continuous variables were compared using ANOVA with post hoc analysis or by Student’s t-test. Mann-Whitney U test was used for covariates with non-normal distribution. Correlations were analyzed using Spearman’s analysis for non-parametric data and Pearson’s analysis for parametric data. A p value ≤ 0.05 was considered significant. All analyses were performed using SPSS (version 14).

## Results

### Patient characteristics

Between 2010 and 2012, 861 cases were screened, 320 cases met enrollment criteria; 181 dengue cases and 35 non-dengue cases were enrolled. The reasons for not being enrolled were: 1) patients already developed clinical signs of shock at the time of screening, 2) failure to obtain informed consent and/or assent, 3) the number of cases exceeded the weekly enrollment limit (6 cases per week). Table 1 shows clinical and laboratory findings on the first day of the study. Dengue cases were classified as 119 DF cases and 12, 28, 21, and 1 cases of DHF Grades I, II, III, and IV, respectively (Table 1). Twenty-three cases were classified as severe dengue (SD) according to 2009 WHO guidelines; all met the DHF case definition. The diagnosis of non-dengue cases were: unspecified viral illness (28 cases), influenza (3 cases), respiratory syncytial virus infection (1 case), pneumonia (1 case), and sinusitis (2 cases). Dengue cases were older than non-dengue febrile illness cases (p = .002, ANOVA) and had higher hematocrit levels on the day of enrollment (p = .005, ANOVA). DHF cases had higher levels of alanine aminotransferase (ALT) and hematocrit and lower albumin and platelet counts compared to DF cases (all at p<0.05, ANOVA). There were no deaths or intensive care unit admissions, and none received vasopressive or inotropic support.

**Table 1:** Clinical characteristics of study participants.

	Diagnosis		
	Non-dengue	DF	DHF
N	35	119	Gr I 12
			Gr II 28
			Gr III 21
			Gr IV 1
M/F	18/17	71/48	34/28
Age, y (SE)	7.74 (.40)	9.78 (.27) ^*a*^	9.48 (.43) ^*a*^
Duration of fever prior to enrollment (days)	4.7 (.27)	4.5 (.10)	4.4 (.15)
2009 classification (SD/D)	N/A	0/119	23/39
Shock (N)			22
Significant bleeding			7
Liver enzyme > 1000			3
Other isolated organ failure			0
AST, IU/ml	48.5 (6)	126 (11)	429 (238)
ALT, IU/ml	23 (4)	61 (6)	145 (59) ^*a*^
Platelet counts, cells/μl	172600 (8314)	115932 (3807) ^*a*^	61177 (4348) ^*a*^ ^,^ ^*b*^
Hematocrit, %	36 (.5)	38 (.4) ^*a*^	41 (.6) ^*a*^ ^,^ ^*b*^
White blood cell counts, cells/μl	4262 (259)	3026 (132) ^*a*^	4230 (384)
Albumin, g/dl	3.9 (.04)	3.9 (.03)	3.5 (.06) ^*a*^ ^,^ ^*b*^
Dengue immune status			
Primary	N/A	14	0
Secondary	N/A	105	62
Dengue serotypes			
D1/D2/D3/D4/unidentified	N/A	34/27/38/2/18	12/24/14/1/11

Fever day and laboratory findings were from the day of study entry. Values represent mean (SE), or number of cases.

^*a*^ Different from non-dengue cases (*P* < .05)

^*b*^ different from DF (*P* < .05).

### Hemodynamic and functional cardiac indices on the first day of study

Table 2 shows the hemodynamic and cardiac functions of dengue cases on the first study day. Cases were classified into DF and DHF. To examine cardiovascular functions in the context of plasma leakage, we further divided DHF cases based on the presence of ultrasonographic evidence of plasma leakage at this time point. Differences were observed between DHF cases that already showed plasma leakage at this time point (n = 36) compared to DHF cases that had not yet developed plasma leakage (n = 24) and DF cases (n = 119). DHF cases with plasma leakage had decreased stroke volume and cardiac index (CI), and elevated systemic vascular resistance (SVR) (p = .001, .003, .003, respectively, Student’s t-test). These indices were not different between DF cases and DHF cases that had not yet developed plasma leakage. Diastolic and systolic cardiac indices also differed between groups. MV-E wave and ejection fraction (EF) were lower in DHF cases with plasma leakage compared to those without (p = .01, and .068) and to DF (p = .001, and .006). There were 6 cases with EF < 56%; all had evidence of plasma leakage. There were no differences in age, sex, and viral serotypes of DHF cases with or without plasma leakage at this time point.

**Table 2:** Laboratory findings and cardiac function measurements on the first study day in subjects with dengue.

		Diagnosis	
	DF	DHF (no leakage)^*a*^	DHF(leakage)
Number	119	24	36
Fever day	.34	.83 (.007)	0 (.013) ^*b*^
Liver enzymes			
AST, IU/ml	126 (11)	142(22)	636(408)
ALT, IU/ml	61 (6)	65(14)	202(102)
Platelet counts, cells/μl	115932 (3807)	86125. (7362)	41638(3014) ^*b*^
Hematocrit, %	38 (.4)	40.0(.78)	41.7 (.84)
Albumin, g/dl	3.99 (.42)	3.8 (.05)	3.3 (.09) ^*b*^
Cardiac enzymes			
Troponin-T (pg/ml)	4.72 (2.9)	5.98 (2.1)	4.9 (.6)
CPK-MB (ng/ml)	11.4 (.6)	10.3 (.5)	11.4 (1.3)
HR, beats/min	91 (1.4)	94 (3.7)	99 (2.6)
Pulse pressure, mm Hg	32.7 (0.6)	32.2(1.8)	22.5(1.3) ^*b*^
Stroke volume (ml)	41.4 (1.3)	43.6 (3.1)	30.0 (2.6) ^*b*^
Cardiac index (L/min/m^2^).	3189 (75)	3173 (857)	2695 (767) ^*c*^
SVR (dynes-s/cm^5^)	1795 (63)	1820 (133)	2478 (184) ^*b*^
Left ventricular systolic functions			
EF, %	68.1 (1.3)	67.6 (1.3)	64.3 (1.2)(p = .068 compared to DHF (no leakage)
Left ventricular diastolic functions			
MV-E (cm/s)	99.9 (6.63)	94.6 (3.8)	75.4 (2.5) ^*b*^
MV-A (cm/s)	56.50(1.34)	54.63 (2.78)	49.73 (1.79)
MV-E/A	2.02(.125)	1.79 (0.8)	1.56 (0/6) ^*c*^
Intravascular volume indicator			
Inferior vena cava diameter, mm	10.22 (.34)	11.25 (.76)	8.42 (.62) ^*c*^

Values represent mean (SE), or number of cases.

^a^ two DHF cases did not have ultrasonography performed on the day of enrollment and were not included in this analysis. Differences between groups were analyzed by ANOVA with post hoc test or by Man Whitney’s test.

^*b*^, ^*c*^ different from DHF without leakage at *P* < .005, and .05 respectively).

The above findings suggest that differences in hemodynamic status and LV functions were associated with plasma leakage and decreased intravascular volume. To examine cardiac functions that are less volume dependent, we performed tissue Doppler imaging (TDI) of the LV. TDI measurements of myocardial excursion at the MV during diastole and systole revealed lower early LV diastolic motion as indicated by lower Ea velocity in DHF with leakage (Table 3). E/Ea ratios were reduced in these patients indicating comparatively lower LV filling pressure. Mean myocardial performance index (MPI) of the LV was higher (indicating poorer cardiac performance) in DHF with leakage compared to the other two groups. The extent of plasma leakage as indicated by the size of pleural effusion as measured on ultrasound examination inversely correlated with early and late LV diastolic wall movement in both the septal and lateral regions (R = -.296,-.158, and-.273 for lateral Ea, lateral Aa, and septal Ea waves, respectively. (P = .001, .027, .001). However, no correlations were observed between the severity of plasma leakage and LV systolic wall movement (septal S wave and lateral S wave).

**Table 3:** Tissue Doppler imaging studies of LV function on the first study day in subjects with dengue.

		Diagnosis	
	DF	DHF (no leakage)^*a*^	DHF(leakage)
Number	119	24	36
Left ventricular systolic functions			
TDI-S’ lat (cm/s)	10.2 (.24)	10.4 (2.4)	10.3 (2.8)
TDI-S’ medial (cm/s)	7.84(.123)	8.14 (1.29)	7.40 (1.6) (.p = 059 compared to DHF (no leakage)
Left ventricular diastolic functions			
TDI-MV-lat-Ea (cm/s)	20.93 (1.11)	18.02 (.83)	15.29 (.56)^*b*^
TDI-MV-lat-Aa (cm/s)	9.00 (.71)	7.40 (.34)	6.70 (.24)
TDI-MV-medial-Ea (cm/s)	13.25 (.23)	12.30 (.34)	11.12(.44)^*b*^
TDI-MV-medial-Aa (cm/s)	6.76 (.17)	7.44(.38)	6.47(.21) ^*b*^
E/Ea-lateral	5.34 (.125)	5.54 (.21)	5.05 (.18)
E/Ea-medial	7.62(.15)	7.91 (.28)	6.85 (.21)^*c*^
LV mean performance index (MPI)	.373 (.01)	.328 (.026)	.413 (.021)^*b*^

Values represent mean (SE), or number of cases.

^a^ two DHF cases did not have ultrasonography performed on the day of enrollment and were not included in this analysis. Differences between groups were analyzed by ANOVA with post hoc test or by Man Whitney’s test.

^*b*^, ^*c*^ different from DHF without leakage at *P* < .05, and .005 respectively).

Comparative analysis of hemodynamic and cardiac functional indices observed on the first day of the study in DHF cases who did not have plasma leakage at that time point to the values of these indices observed at the time of subsequent plasma leakage demonstrated evidence of volume contraction and worsening cardiac function (S1 Table and S2 Table). However, the differences were comparatively smaller than those between DHF with or without leakage at the study enrollment. This may be due to the close clinical monitoring and timely fluid intervention in these cases.

High proportions of non-dengue and dengue cases demonstrated abnormal TDI parameters including low amplitudes of S waves and Ea waves indicating defects in systolic and diastolic movement of the left ventricle (Table 4 and S3 Table). Forty-two to sixty-three percent of dengue patients had low medial or lateral S waves compared to 21–33% in non-dengue cases but these differences were not statistically significant. However, the proportions of dengue cases with low Ea wave amplitudes were higher than non-dengue cases (p = .001) with the highest frequency found in DHF cases who already developed plasma leakage. The percentages of cases with abnormally high E/Ea ratio, which indicates elevated LV filling pressure, were higher in DHF cases that had not developed plasma leakage (64%) compared to DF (41%, p = .049) and DHF with plasma leakage (22%, p = .001).

**Table 4:** Frequencies of cases with abnormal TDI findings on the first day of the study.

	Clinical classification (%)^*a*^			
TDI parameters	Non-dengue	DF	DHF without leakage	DHF with leakage
Low S medial	33	47	45	66
Low S lateral	21	46	42	44
Low Ea medial	30	52 ^*b*^	59^*b*^	80^*b*^ ^,^ ^*c*^
Low Ea lateral	14	28	42 ^*d*^	64 ^*d*^ ^,^ ^*e*^
Low Aa medial	18	25	27	36
Low Aa lateral	3 ^*f*^ ^,^ ^*g*^	21	29	39^*h*^
High E/Ea	44	41	64 ^*i*^ ^,^ ^*j*^ ^,^ ^*k*^	22

*a*, Percentages of cases with abnormal TDI findings were calculated from the numbers of records with abnormal tracings divided by the numbers of all records with analyzable tracings and multiplied by one hundred. The numbers of records with abnormal tracings and the number of analyzable records for each parameters and diagnostic category are shown in S3 Table. Two DHF cases did not have ultrasonography performed on the day of enrollment and were not included in this analysis.

*b*, different from non-dengue cases (p = .033)

*c*, different from DF (p = .001)

*d*, different from non-dengue (p = .001)

*e*, different from DF (p = .001)

*f*, different from DF (p = .013)

*g*, different from DHF with or without leakage, (p = .004)

*h*, different from DF (p = .045)

*i*, different from non-dengue (p = .045)

*j*, different from DF (p = .049)

*k*, different from DHF with leakage (p = .001)

### Changes in hemodynamic status, cardiac function, and plasma leakage over the course of illness

**Fig 1:**
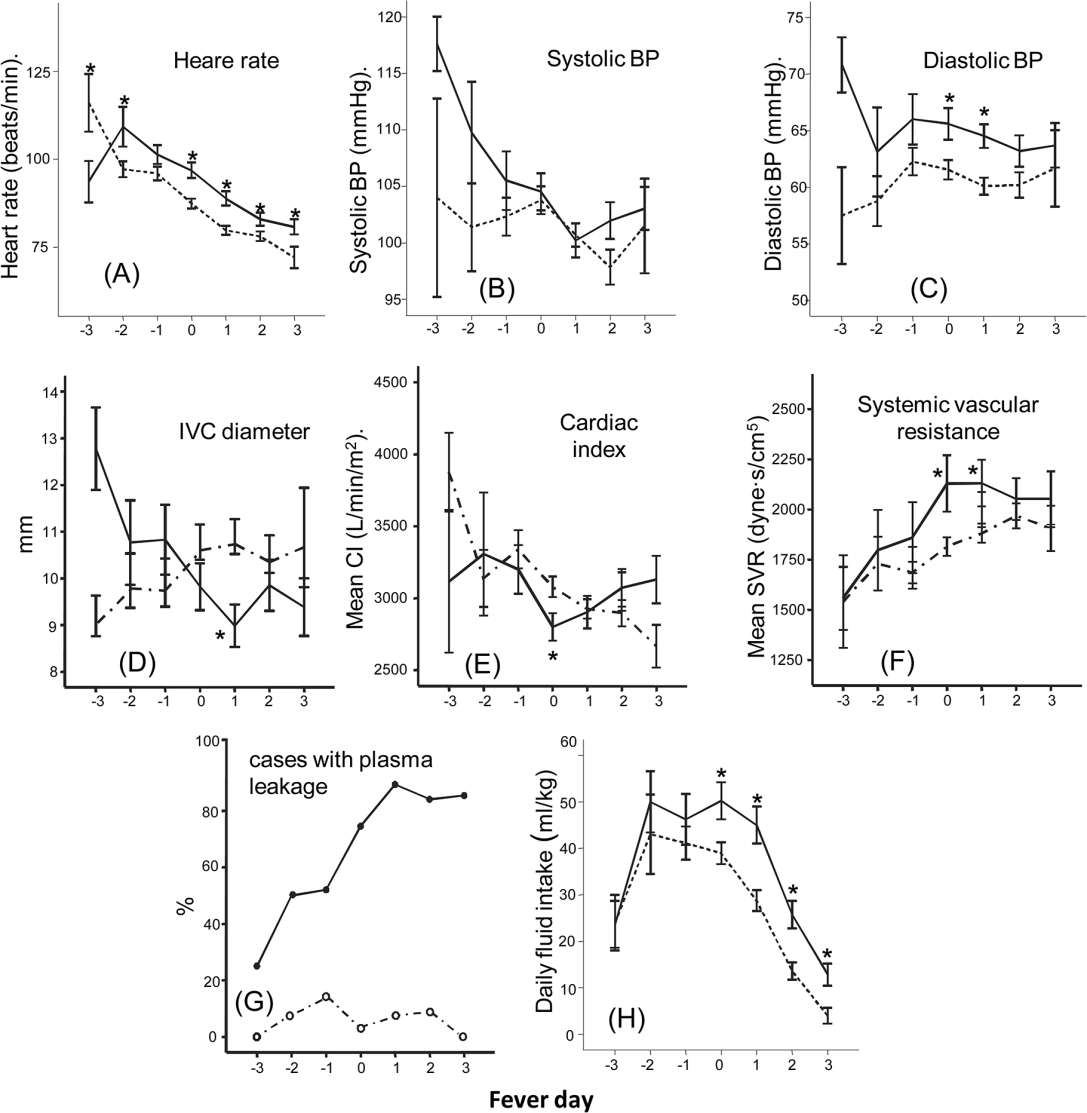
Hemodynamic status and plasma leakage in dengue by fever day. Heart rate (A), systolic blood pressure (B), diastolic blood pressure (C), inferior vena cava diameter (D), mean cardiac index (E), systemic vascular resistance (F), frequencies of cases with a pleural effusion or ascites (G), and daily fluid intake (H) of dengue fever (dotted line) and dengue hemorrhagic fever cases (solid line) over the course of the illness. Fever day 0 denotes day of defervescence. * different from dengue fever cases (*P* < .05). The numbers of cases were: fever-day -3 (DF 4, DHF 5), fever-day-2 (DF 13, DHF 9), fever-day-1(DF 43, DHF 26), fever-day-0(DF 104, DHF 56), fever-day+1(DF 119, DHF 62), fever-day+2(DF 70, DHF 60), fever-day+3(DF 13, DHF 38).

Fig 1 shows the patterns of hemodynamic and volume indices and fluid intake over the course of the illness of DF and DHF cases. DHF cases had higher heart rate compared to DF early in the course of illness and this persisted throughout the acute illness (A). There was no difference in systolic blood pressure (B), but mean diastolic pressures were higher in DHF cases around the day of fever resolution (fever day 0) and shortly thereafter (C). This coincided with decreased intravascular volume as indicated by a decrease in the average IVC diameter in DHF cases (D). DHF cases had lower CI (E) at defervescence (fever day 0) and increased SVR (F) on fever day 0 and +1 compared to DF cases. Ultrasonography revealed a gradual increase in the percentage of cases with plasma leakage, with peak incidence on fever day +1 (G). Patients with DHF also received more fluid compared to those with DF starting on fever day 0 and afterward.

**Fig 2:**
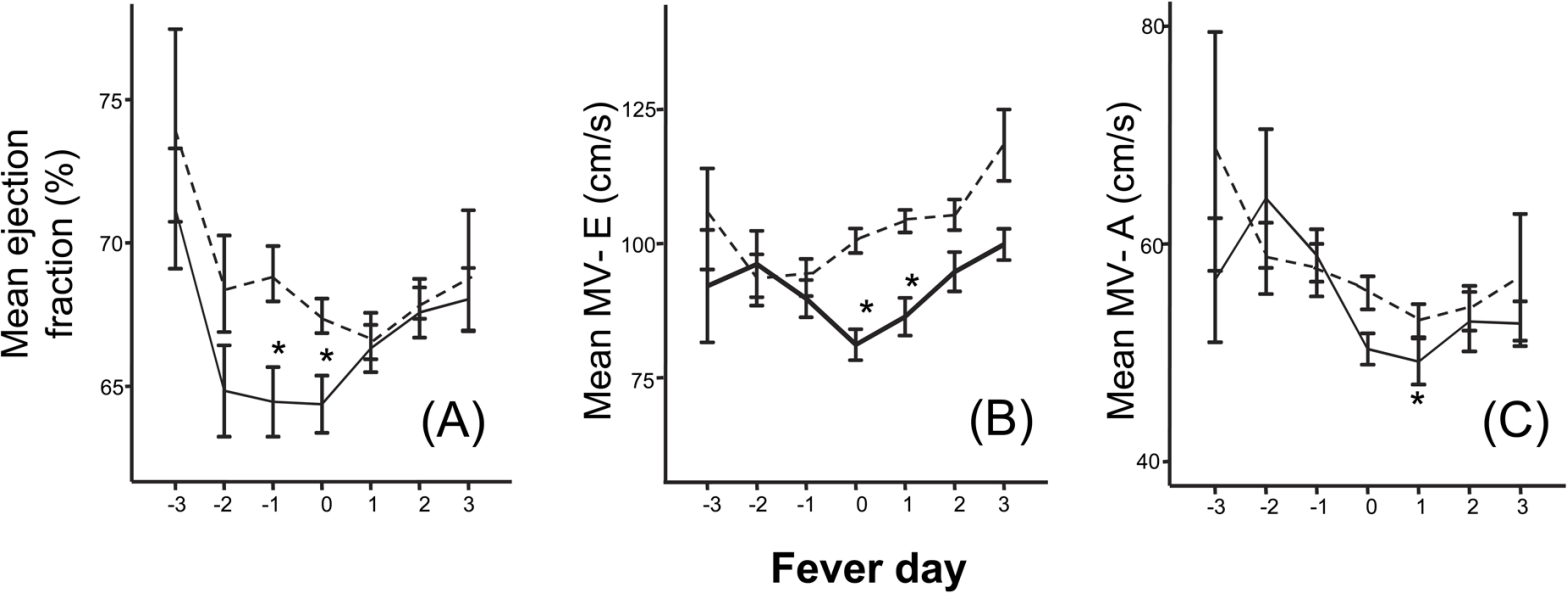
Left ventricular functions in dengue by fever day. Ejection fraction (A), early diastolic LV inflow flow (B), and late diastolic LV inflow (C) of dengue fever (dotted line) and dengue hemorrhagic fever cases (solid line) over the course of the illness. Fever day 0 denotes day of defervescence. * different from dengue fever (*P* < .05). The numbers of cases were: fever-day -3 (DF 4, DHF 5), fever-day-2 (DF 13, DHF 9), fever-day-1(DF 43, DHF 26), fever-day-0(DF 104, DHF 56), fever-day+1(DF 119, DHF 62), fever-day+2(DF 70, DHF 60), fever-day+3(DF 13, DHF 38).

Evaluation of LV systolic and diastolic functions revealed lower EFs (Fig 2A) on fever day -1 and 0, and lower early component of LV inflow (MV-E wave) on fever days 0 and +1 (Fig 2B) in DHF compared to DF. The late component LV inflow was also decreased in DHF on fever day +1 (Fig 2C). Seventeen dengue cases had at least one abnormal EF (<56%) detected during the illness. They represented 7%, 9%, 15%, and 32% of DF, DHF grade I, II, and DHF Gr III/IV cases, respectively. The relative frequencies of abnormal EFs were higher in DHF grade III/IV compared to DHF grade I/II and DF (p < .001, Chi-square). The majority of cases (67%) had low EF detected on only a single day, usually fever day 0 or +1. The EFs on the day of discharge were improved compared to the lowest EF. Taken together, this indicates that changes in hemodynamic status in DHF were temporally associated with plasma leakage and were characterized by a compensatory autonomic response to contracted intravascular volume which was corrected by fluid replacement.

### Tissue Doppler assessment of cardiac wall motion

**Fig 3:**
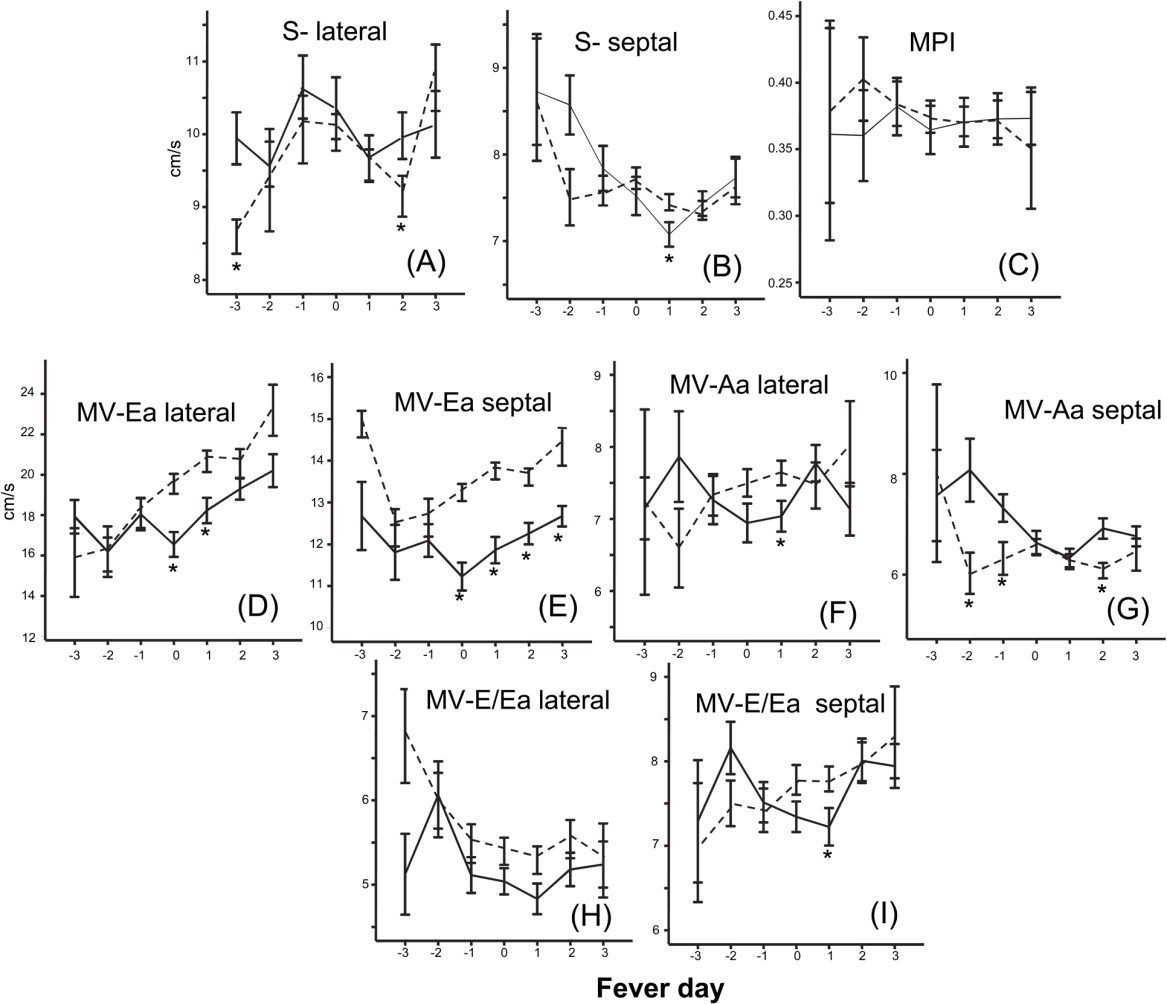
Tissue Doppler image of LV functions by fever day. Left ventricular lateral (A) and septal S wave (B), LV mean performance index (MPI) (C), lateral (D) and septal (E) annulus movement during early diastole, lateral (F) and septal (G) annulus movement during late diastole, and lateral (H) and medial (I) E/Ea ratios of dengue fever (dotted line) and dengue hemorrhagic fever (solid line) cases over the course of the illness. Fever day 0 denotes day of defervescence. * different from DF (*P* < .05). The numbers of cases were: fever-day -3 (DF 4, DHF 5), fever-day-2 (DF 13, DHF 9), fever-day-1(DF 43, DHF 26), fever-day-0(DF 104, DHF 56), fever-day+1(DF 119, DHF 62), fever-day+2(DF 70, DHF 60), fever-day+3(DF 13, DHF 38).

TDI of the MV showed slower septal movement during systole in DHF compared to DF on fever day +1 (p = .031) (Fig 3B). Early and late diastolic LV annulus motions (Ea and Aa wave) were lower in DHF cases compared to DF cases at the end of the febrile period and after (Fig 3D, 3E and 3F). The decreased early diastolic annulus motion was more pronounced in the septal area. The E/Ea ratio, which indicates LV filling pressure, was not different between DHF and DF cases on most days except on fever day +1 when the average E/Ea ratio was significantly lower in DHF cases (I).

**Fig 4:**
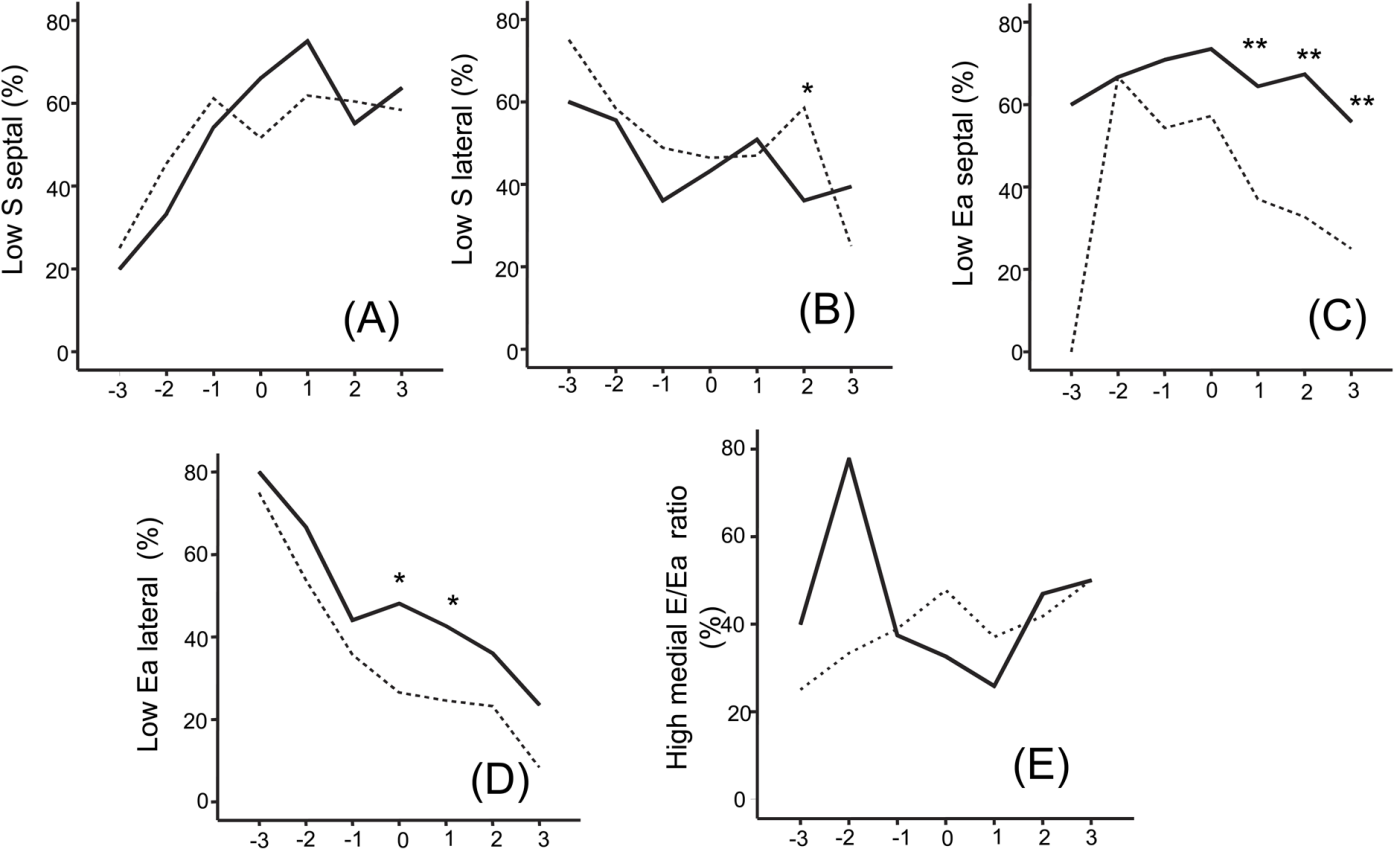
Frequencies of abnormal TDI during the course of the illness. Percentages of DF (dotted lines) and DHF cases (solid lines) with low septal and lateral S wave (A, B) and septal and lateral Ea wave (C, D) and high E/Ea ratios (E) determined based on published age-specific normal values (21). * different from DF (*P* < .05).

Twenty to seventy percent of dengue cases had abnormally low diastolic and systolic wall motion during the course of the illness (Fig 4A–4D). The frequencies of abnormally low diastolic wall motion were higher in DHF compared to DF on fever day 0 and after (Fig 4C and 4D). Elevated E/Ea ratios were found in both DF and DHF cases with similar frequencies. None had E/Ea ratio above 15.

**Fig 5:**
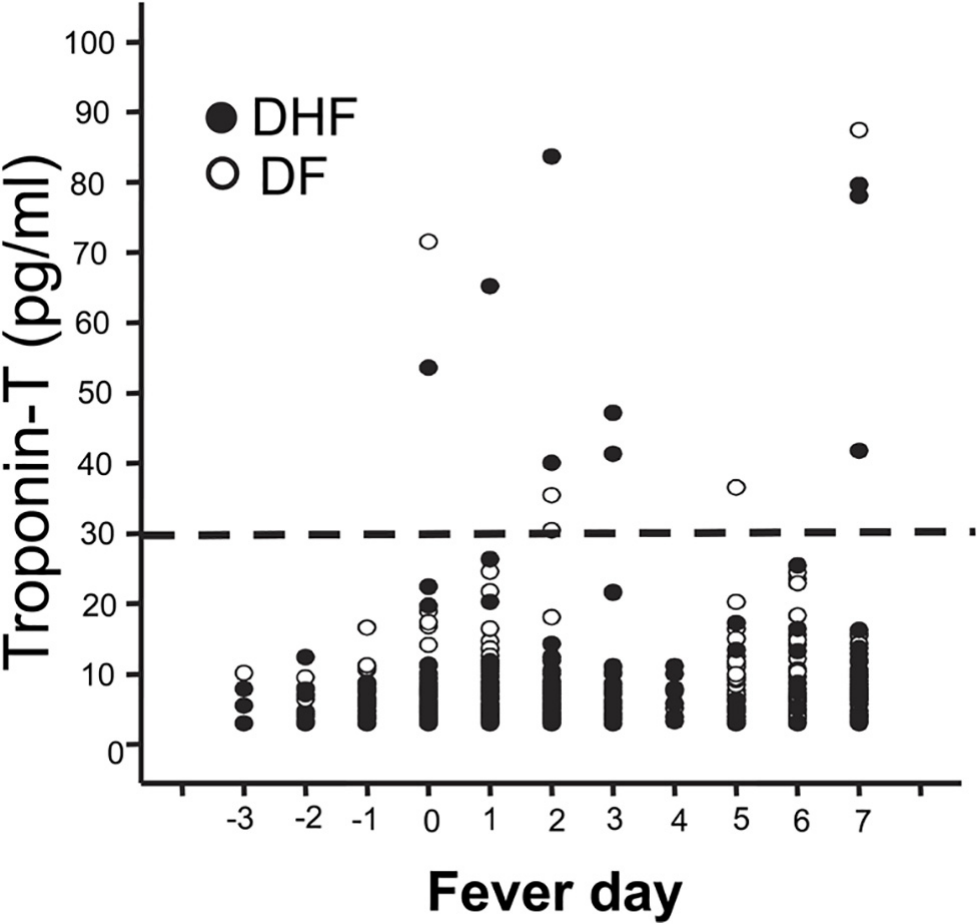
Plasma troponin-T levels in individual DF (white circles) or DHF cases (black circles) during the course of the illness. The horizontal line indicates a cut-off for an abnormal value at 30 pg/ml.

Troponin and CPK-MB levels were similar between dengue and non-dengue cases on the day of study enrollment. Plasma levels of cardiac troponin-T were low to non-detectable in most cases with comparable levels found in DF and DHF cases throughout the acute illness period. Consistent with this finding, the mean levels of CPK-MB were similar between DF and DHF. However, 14.5% of DHF cases had troponin-T levels >30 ng/L at any time point compared to 5% in DF (p = 0.028, Chi-square test). All of these cases had only one sample with elevated troponin-T levels during the course of illness. These elevated troponin levels were detected later in the course of the illness (Fig 5). There were no cases with clinical heart failure or conductive defects consistent with clinical myocarditis.

Pericardial effusion was detected in 7 DHF cases. The size of the effusion was minimal in 6 cases and substantial in 1 case. Sinus bradycardia was documented in 1 DF case. Four cases of mild mitral regurgitation (2 DF and 2 DHF) and 2 DF cases with tricuspid regurgitation were found. These findings were transient and not clinically significant.

Twelve patients (4 DF, 8 DHF) had a follow up examination performed at early convalescence (< 1 week after discharge). Trends toward improvement were found for EF and LV inflow at the MV (MV-E) and diastolic medial LV wall movement (medial Ea wave) in DHF cases at convalescence compared to fever day +1 (Table 5).

**Table 5:** Comparison between cardiac function at fever day +1 and early convalescence.

	DF					DHF				
	feverday+1		early convalescence			feverday+1		early convalescence		
	average	(SEM)	average	(SEM)	^*a*^ *P*	average	(SEM)	average	(SEM)	^*a*^ *P*
EF (%)	70.88	4.29	71.03	1.01	0.967	66.46	2.58	72.48	1.23	0.075
MV-E (cm/s)	99.90	11.50	98.35	11.02	0.414	83.63	8.86	98.93	6.32	0.049
MV Lat Ea (cm/s)	21.68	1.53	20.03	1.77	0.472	18.01	1.94	22.90	2.18	0.184
MV Med Ea (cm/s)	13.98	0.55	14.08	1.41	0.937	11.73	0.61	14.18	0.36	0.014
Lat E/Ea ratio	4.57	0.27	4.99	0.68	0.442	4.94	0.25	4.50	0.20	0.538
Med E/Ea ratio	7.24	1.04	7.41	1.49	0.858	7.11	0.16	7.0	0.09	0.794

^a^ *P* value comparing values at fever day +1 and early convalescence (Student’s paired t-test).

## Discussion

Much of the literature on cardiac manifestations in dengue consists of case reports and small case series. More recent prospective studies have reported varied incidence of abnormal cardiac findings from approximately 15–27% for myocarditis [8, 24, 25] and up to 40% for functional abnormalities[26]. Cardiac performance and hemodynamic status are affected by intravascular volume, cardiac functions and autonomic response. The present study extends our knowledge of cardiac involvement in dengue in several ways. We obtained daily echocardiographic studies over the critical phase of illness in a cohort that included both milder and more severe illness, and analyzed cardiac function data in conjunction with serial assessments of intravascular volume status and plasma leakage. The data show that cardiac functional abnormalities are common in dengue and correlate with disease severity. However, these abnormalities were transient, did not require specific treatment, and were not accompanied by evidence of structural damage to the myocardium.

Our study found that cardiac functional abnormalities were related to the severity of plasma leakage. Decreased EF, CI, LV diastolic inflow, and the elevated SVR in DHF are therefore likely to be affected by contracted intravascular volume. TDI indices, which are less preload dependent [27, 28], also differed between dengue and non-dengue cases, and between DF and DHF. This was most evident in decreased LV wall movement during early diastole indicating a relaxation defect. The higher frequencies of cases with decreased LV early diastolic wall movement in DF compared to non-dengue cases suggests a dengue specific process independent of plasma leakage. However, the correlation between abnormal LV diastolic movement and the extent of plasma leakage among dengue cases suggests that plasma leakage may also directly contribute to this functional abnormality by reducing intravascular volume. Alternatively, plasma leakage may be a correlate of the disease mechanism that affects cardiac function. The relatively similar E/Ea ratios in DF and DHF despite lower E in DHF reflect this diastolic defect. The pathology underlying this finding is unknown. Nevertheless, the abnormal LV relaxation may further compromise LV filling. Decreased LV relaxation may also contribute to high LV filling pressure when intravascular volume has been restored by intravenous fluid treatment and by reabsorption of effusion fluid, and may lead to the pulmonary edema observed in some DHF cases. The prevalence of subclinical diastolic dysfunction has been reported to increase with age, in individuals with hypertension, LV hypertrophy, and diabetes [29–31]. This has important implications for clinical care considering the shift in the demographics of dengue cases to adults who may have these common co-morbidities. Further studies in adults with dengue will be needed to address this question.

Fatal dengue associated with myocardial injury and inflammation has been reported. In a study of adult and pediatric cases from Brazil, the incidence of myocarditis on the basis of clinical diagnosis or elevated biomarkers was approximately 15% [24]. A subset of these cases had abnormal echocardiography or MRI [8, 24]. Viral antigen has been detected in cardiac myocytes, monocytes, and endothelial cells by immunostaining [7, 8, 32]. Contrary to these reports, we did not observe cases with myocarditis based on clinical information and cardiac enzymes. Our findings are consistent with other reports in pediatric dengue cases in Southeast Asia [26, 33]. The difference in the incidence of myocarditis in various reports may be related to host genetic factors and locally circulating viruses, which may influence tissue tropism and host inflammatory responses. In addition, different temporal patterns of circulating serotypes may lead to distinct dengue immune status that predisposes individuals for more severe cardiac manifestation during a secondary infection. However, differences in age groups, co-morbidities, and study design may also contribute to differences in manifestations and incidence Although sustained elevated enzyme levels were not found, transient increased levels were found in some cases, particularly in DHF, which may indicate subtle myocardial injury in these cases.

Cardiac dysfunction has been reported in other viral hemorrhagic fevers such as Crimean-Congo hemorrhagic fever and Puumala virus infection [34–36]. The patterns of cardiac involvement are similar to dengue and are characterized by transient functional impairment and normal cardiac enzyme levels. Another similarity is more severe involvement in adults. These findings suggest common pathophysiologic pathways including endothelial cell activation, perturbation of vascular permeability, and cardiac muscle cell dysfunction. Cardiac dysfunction is also common in sepsis in which TNF-α and nitric oxide are considered to be involved [37, 38]. These mediators have been reported to be altered in dengue as well [39–41]. These mediators may alter cardiac function and hemodynamic status by their permeability enhancing effects, which lead to decreased preload, as well as direct effects on cardiac myocytes and on vascular resistance. The relative contribution of these mediators will need to be further addressed in relevant animal models.

Declining heart rate during defervescence has been observed in dengue and attributed to increased parasympathetic activity[42]. Benign bradyarrhythmias and ectopic beats have been reported in patients with DENV infection following defervescence and were not related to disease severity[43]. The prevalence of cardiac arrhythmia in the present study was lower than previously reported. We did not perform continuous electrocardiographic monitoring and therefore some cases might have been missed. Another limitation was the lack of adult cases that may exhibit distinct cardiac manifestations. Due to the conservative use of intravenous fluid there were no cases with pulmonary edema and fluid overload in this series. However, the relatively normal or elevated E/Ea ratio in DHF cases in the presence of contracted intravascular volume suggests that diastolic dysfunction may predispose some DHF cases to pulmonary edema with aggressive administration of intravenous fluid.

In summary, functional cardiac abnormalities in dengue involved both systolic and diastolic functions and correlated with the severity of plasma leakage. Cardiac structural changes such as infarction and myocarditis were not likely the underlying mechanism. Cardiac dysfunction was transient and did not require specific treatment. Decreased LV wall diastolic movement may contribute to abnormal LV filling and the predisposition for pulmonary edema in DHF. These findings underscore the importance of vigilance in fluid management and hemodynamic status monitoring in the treatment of dengue.

## Supporting Information

S1 Checklist STROBE checklist. (DOCX)

S1 Table Laboratory findings and cardiac function measurements at study enrollment of DHF cases without plasma leakage and the subsequent findings on day of plasma leakage. (DOCX)

S2 Table Tissue Doppler imaging values at study enrollment of DHF cases without plasma leakage and the subsequent findings on day of plasma leakage. (DOCX)

S3 Table Numbers of cases with abnormal TDI findings on the first day of the study. (DOCX)
