# Supplementary material for: Evaluation of Cardiac Involvement in Children with Dengue by Serial Echocardiographic Studies
Source: PLoS Negl Trop Dis. 2015 Jul 30;9(7):e0003943. doi: 10.1371/journal.pntd.0003943 (PMC4520477; doi:10.1371/journal.pntd.0003943)
Supplement: S1 Checklist — (DOCX) [file pntd.0003943.s001.docx]

STROBE Statement—Checklist of items that should be included in reports of ***cohort studies***

|  | Item No | Recommendation |
| --- | --- | --- |
| **Title and abstract** | 1 | (*a*) Indicate the study’s design with a commonly used term in the title or the abstract  **The abstract states “prospective in-patient cohort of suspected dengue cases.”** |
|  |  | (*b*) Provide in the abstract an informative and balanced summary of what was done and what was found  **This was provided in the abstract.** |
| Introduction | | |
| Background/rationale | 2 | Explain the scientific background and rationale for the investigation being reported  **The introduction states: “Severe plasma leakage can lead to shock (dengue shock syndrome). Although the principal mechanism of shock is due to decreased intravascular volume, abnormal cardiac functions may contribute to cardiovascular compromise.”** |
| Objectives | 3 | State specific objectives, including any prespecified hypotheses  **The introduction states: “To further our understanding of cardiac involvement in dengue, we undertook serial studies of cardiac function in dengue cases.”** |
| Methods | | |
| Study design | 4 | Present key elements of study design early in the paper  **This was presented early in the Abstract section under the sub-section Methods and Principal finding: We characterized the incidence and changes in cardiac function in a prospective in-patient cohort of suspected dengue cases by serial echocardiography.”** |
| Setting | 5 | Describe the setting, locations, and relevant dates, including periods of recruitment, exposure, follow-up, and data collection  **This was described in the Methods section under the sub-sections “Patients” as follows ”Children less than 15 years of age who were hospitalized for suspected dengue at Queen Sirikit National Institute of Child Health (QSNICH) in 2010 to 2012 were enrolled.”** |
| Participants | 6 | (*a*) Give the eligibility criteria, and the sources and methods of selection of participants. Describe methods of follow-up.  **This was described in the Methods section under the sub-sections “Patients” as follows ”Children less than 15 years of age who were hospitalized for suspected dengue at Queen Sirikit National Institute of Child Health (QSNICH) in 2010 to 2012 were enrolled. Criteria for suspected dengue included cases with febrile illness without an obvious focal source of infection and with compatible laboratory findings including leucopenia or thrombocytopenia.** **Patients with chronic hematologic or immunologic conditions were excluded.”** |
|  |  | (*b*) For matched studies, give matching criteria and number of exposed and unexposed  **Not applicable.** |
| Variables | 7 | Clearly define all outcomes, exposures, predictors, potential confounders, and effect modifiers. Give diagnostic criteria, if applicable  **The variables were interpreted in relation to clinical diagnosis. This was described in the Methods section under the sub-section “Patients” for case clinical classifications. The references for the clinical classification systems were provided.** |
| Data sources/ measurement | 8* | For each variable of interest, give sources of data and details of methods of assessment (measurement). Describe comparability of assessment methods if there is more than one group  **This was described in the Methods section under the sub-section “Patients.” For clinical classification. Cardiac enzyme level measurement was described in the Methods section under the sub-section “Cardiac enzymes.” Echocardiograhic variables were described in the Methods section under the sub-section “Echocardiography.”** |
| Bias | 9 | Describe any efforts to address potential sources of bias  **This was described in the Methods section under the following sub-sections: 1) “Patients.” ; Case classification was performed after the completion of the study irrespective of diagnosis made during admission by investigators not involved in patient care., 2) “Cardiac enzymes”. All tests were performed in batch after study completion and without the knowledge of clinical diagnosis, 3) “Echocardiography”; All measurements were obtained on a daily basis without the knowledge of the clinical diagnosis and the diagnostic laboratory results.** |
| Study size | 10 | Explain how the study size was arrived at  **This is an exploratory study; we targeted the sample size of each continuous variable to be at about 25 for each diagnostic group so that the distribution of the data could be analyzed in order to appropriately apply either parametric or non-parametric tests. The study size was also dictated by logistics as indicated in the “Patient characteristics” section under the “Result” section.** |
| Quantitative variables | 11 | Explain how quantitative variables were handled in the analyses. If applicable, describe which groupings were chosen and why  **All quantitative variables were included in the analysis. They were analyzed in the context of final clinical classification (diagnosis).** |
| Statistical methods | 12 | (*a*) Describe all statistical methods, including those used to control for confounding  **Basic statistical methods were used for analysis as summarized in the “Statistical Methods” sub-section.** |
|  |  | (*b*) Describe any methods used to examine subgroups and interactions  **Basic statistical methods were used for analysis as summarized in the “Statistical Methods” sub-section.** |
|  |  | (*c*) Explain how missing data were addressed  **There were minimal and not included in any analyses.** |
|  |  | (*d*) If applicable, explain how loss to follow-up was addressed  **Not applicable.** |
|  |  | (*e*) Describe any sensitivity analyses  **Not performed.** |
| Results | | |
| Participants | 13* | (a) Report numbers of individuals at each stage of study—eg numbers potentially eligible, examined for eligibility, confirmed eligible, included in the study, completing follow-up, and analysed  **These were included in the “Patient Characteristics” section.** |
|  |  | (b) Give reasons for non-participation at each stage  **These were included in the “Patient Characteristics” section.** |
|  |  | (c) Consider use of a flow diagram  **Not applicable.** |
| Descriptive data | 14* | (a) Give characteristics of study participants (eg demographic, clinical, social) and information on exposures and potential confounders  **See Table 1.** |
|  |  | (b) Indicate number of participants with missing data for each variable of interest  **These were included in S3_Table.** |
|  |  | (c) Summarise follow-up time (eg, average and total amount)  **Not applicable.** |
| Outcome data | 15* | Report numbers of outcome events or summary measures over time  **This is reported in the Results section, table 1.** |
| Main results | 16 | (*a*) Give unadjusted estimates and, if applicable, confounder-adjusted estimates and their precision (eg, 95% confidence interval). Make clear which confounders were adjusted for and why they were included  **This is provided in the Results section.** |
|  |  | (*b*) Report category boundaries when continuous variables were categorized  **This is provided in the Results section as standard error of the mean (SEM).** |
|  |  | (*c*) If relevant, consider translating estimates of relative risk into absolute risk for a meaningful time period  **Not applicable.** |
| Other analyses | 17 | Report other analyses done—eg analyses of subgroups and interactions, and sensitivity analyses  **Relevant further analyses are presented in the Results section.** |
| Discussion | | |
| Key results | 18 | Summarise key results with reference to study objectives  **These were summarized in the first paragraph in the Discussion section.** |
| Limitations | 19 | Discuss limitations of the study, taking into account sources of potential bias or imprecision. Discuss both direction and magnitude of any potential bias  **Relevant study limitations are described in the last paragraph in the Discussion section.** |
| Interpretation | 20 | Give a cautious overall interpretation of results considering objectives, limitations, multiplicity of analyses, results from similar studies, and other relevant evidence  **This is provided in the Discussion section.** |
| Generalisability | 21 | Discuss the generalisability (external validity) of the study results  **This is mentioned in the Discussion section in that the study results differ from other studies due to differences in age groups, co-morbidities, and study design.** |
| Other information | | |
| Funding | 22 | Give the source of funding and the role of the funders for the present study and, if applicable, for the original study on which the present article is based  **The funding sources were mentioned.** |

*Give information separately for exposed and unexposed groups.

**Note:** An Explanation and Elaboration article discusses each checklist item and gives methodological background and published examples of transparent reporting. The STROBE checklist is best used in conjunction with this article (freely available on the Web sites of PLoS Medicine at http://www.plosmedicine.org/, Annals of Internal Medicine at http://www.annals.org/, and Epidemiology at http://www.epidem.com/). Information on the STROBE Initiative is available at http://www.strobe-statement.org.
