# Supplementary material for: Evaluation of Cardiac Involvement in Children with Dengue by Serial Echocardiographic Studies
Source: PLoS Negl Trop Dis. 2015 Jul 30;9(7):e0003943. doi: 10.1371/journal.pntd.0003943 (PMC4520477; doi:10.1371/journal.pntd.0003943)
Supplement: S1 Table — (DOCX) [file pntd.0003943.s002.docx]

**Supplemental Table 1**

Laboratory findings and cardiac function measurements at study enrollment of DHF cases without plasma leakage and the subsequent findings on day of plasma leakage.

|  | Time of measurement | |  |  |
| --- | --- | --- | --- | --- |
|  | At study enrollment | On the day of plasma leakage | |  |
| Albumin, g/dl | 3.8 (.05) | 3.32(.1)*^a^* | |  |
| HR, beats/min | 92 (3.7) | 91 (3.2) | |  |
| Pulse pressure, mm Hg | 32.2(1.8) | 28.75(1.6)*^b^* | |  |
| Stroke volume (ml) | 43.6 (3.1) | 35.9 (2.7) *^b^* | |  |
| Cardiac index (L/min/m^2^). | 3173 (174) | 2767 (167) *^b^* | |  |
| SVR (dynes-s/cm^5^) | 1820 (133) | 2005 (166) | |  |
| Left ventricular systolic functions | | | | |
| EF, % | 67.6 (1.3) | 62.5 (2.9) | |  |
| Left ventricular diastolic functions | | | | |
| MV-E (cm/s) | 94.6 (3.8) | 86.9 (4.13) *^c^* | |  |
| MV-A (cm/s) | 54.63 (2.78) | 50.53 (2.12) | |  |
| MV-E/A | 1.79 (0.8) | 1.77 (0.9) | |  |
| Intravascular volume indicator | | | | |
| Inferior vena cava diameter, mm | 11.25 (.76) | 8.5 (.72) *^b^* | |  |

Values represent men (SE), or number of cases. Differences between the two time points were analyzed by paired T-test. *^a^*, *^b^* different from the values at study enrollment at *P* < .005, and .05 respectively), *^c^* (*P* = .081).
