# Supplementary material for: Evaluation of Cardiac Involvement in Children with Dengue by Serial Echocardiographic Studies
Source: PLoS Negl Trop Dis. 2015 Jul 30;9(7):e0003943. doi: 10.1371/journal.pntd.0003943 (PMC4520477; doi:10.1371/journal.pntd.0003943)
Supplement: S2 Table — (DOCX) [file pntd.0003943.s003.docx]

**Supplemental Table 2**

Tissue Doppler Imaging values at study enrollment of DHF cases without plasma leakage and the subsequent findings on day of plasma leakage.

|  | Time of measurement | | |  |
| --- | --- | --- | --- | --- |
|  | | At study enrollment | On the day of plasma leakage |  |
| Left ventricular systolic functions | | | | |
| TDI-S’ lat (cm/s) | | 10.4 (2.4) | 9.1 (2.35) |  |
| TDI-S’ medial (cm/s) | | 8.14 (1.29) | 6.93 (1.21)*^a^* |  |
| Left ventricular diastolic functions | | | | |
| TDI-MV-lat-Ea (cm/s) | | 18.02 (.83) | 18.32 (1.17) |  |
| TDI-MV-lat-Aa (cm/s) | | 7.40 (.34) | 6.84 (.37) |  |
| TDI-MV-medial-Ea (cm/s) | | 12.30 (.34) | 11.66(.62) |  |
| TDI-MV-medial-Aa (cm/s) | | 7.44(.38) | 5.85(.28) *^a^* |  |
| E/Ea-lateral | | 5.54 (.21) | 5.08 (.19) |  |
| E/Ea-medial | | 7.91 (.28) | 7.73 (.19) |  |
| LV mean performance index (MPI) | | .328 (.026) | .33 (.03) |  |

Values represent mean (SE), or number of cases. Differences between the two time points were analyzed by paired T-test. *^a^*, *^b^* different from the values at study enrollment at *P* < .005, and .05 respectively).
