# Supplementary material for: Evaluation of Cardiac Involvement in Children with Dengue by Serial Echocardiographic Studies
Source: PLoS Negl Trop Dis. 2015 Jul 30;9(7):e0003943. doi: 10.1371/journal.pntd.0003943 (PMC4520477; doi:10.1371/journal.pntd.0003943)
Supplement: S3 Table — (DOCX) [file pntd.0003943.s004.docx]

**Supplemental table 3**

Numbers of cases with abnormal TDI findings on the first day of the study.

|  | Clinical classification  N *^a^*/N *^b^* | | | |
| --- | --- | --- | --- | --- |
| TDI parameters | Non-dengue | DF | DHF without leakage | DHF with leakage |
| Low S medial | 11/33 | 51/109 | 10/22 | 24/36 |
| Low S lateral | 7/34 | 53/114 | 10/24 | 16/36 |
| Low Ea medial | 10/33 | 56/108 | 13/22 | 29/36 |
| Low Ea lateral | 5/35 | 32/116 | 10/24 | 23/36 |
| Low Aa medial | 6/33 | 27/108 | 6/22 | 13/36 |
| Low Aa lateral | 1/35 | 24/116 | 7/24 | 14/36 |
| High E/Ea | 15/34 | 44/108 | 14/22 | 8/36 |

^a^ numbers of records with abnormal values, ^b^ numbers of analyzable records.
